# Improper inhaler technique is associated with poor asthma control and frequent emergency department visits

**DOI:** 10.1186/1710-1492-9-8

**Published:** 2013-03-06

**Authors:** Hamdan AL-Jahdali, Anwar Ahmed, Abdullah AL-Harbi, Mohd Khan, Salim Baharoon, Salih Bin Salih, Rabih Halwani, Saleh Al-Muhsen

**Affiliations:** 1 Department of Medicine, Pulmonary Division-ICU, King Saud bin Abdulaziz University for Health Sciences, Riyadh, Saudi Arabia; 2 Department of Epidemiology and Biostatistics, College of Public Health and Health Informatics, King Saud bin Abdulaziz University for Health Sciences, Riyadh, Saudi Arabia; 3 Asthma Research Chair and Prince Naif Center for Immunology Research, Department of Pediatrics, College of Medicine, King Saud University, Riyadh, Saudi Arabia; 4 King Saud University for Health Sciences, Head of Pulmonary Division, Medical Director of Sleep Disorders Center, King Abdulaziz Medical City, Riyadh, Saudi Arabia

**Keywords:** Asthma control, Inhaled corticosteroid, Emergency department, Inhaler devices, Asthma education

## Abstract

**Background:**

Uncontrolled asthma remains a frequent cause of emergency department (ED) visits and hospital admissions. Improper asthma inhaler device use is most likely one of the major causes associated with uncontrolled asthma and frequent ED visits.

**Objectives:**

To evaluate the inhaler technique among asthmatic patients seen in ED, and to investigate the characteristics of these patients and factors associated with improper use of inhaler devices and its relationship with asthma control and ED visits.

**Methods:**

A cross-sectional study of all the patients who visited the ED with bronchial asthma attacks over a 9-month period was undertaken at two major academic hospitals in Saudi Arabia. Information was collected about demographic data and asthma management and we assessed the inhaler techniques for each patient using an inhaler technique checklist.

**Results:**

A total of 450 asthma patients were included in the study. Of these, 176(39.1%) were males with a mean age of 42.3 ±16.7 years and the mean duration of asthma was 155.9 ± 127.1 weeks. The improper use of asthma inhaler devices was observed in 203(45%) of the patients and was associated with irregular clinic follow-ups (p = 0.0001), lack of asthma education (p = 0.0009), uncontrolled asthma ACT (score ≤ 15) (p = 0.001), three or more ED visits (p = 0.0497), and duration of asthma of less than 52 weeks (p = 0.005). Multiple logistic regression analysis revealed that a lack of education about asthma disease (OR =1.65; 95% CI: 1.07, 2.54) or a lack of regular follow-up (OR =1.73; 95% CI: 1.08, 2.76) was more likely to lead to the improper use of an asthma inhaler device.

**Conclusion:**

Improper asthma inhaler device use is associated with poor asthma control and more frequent ED visits. We also identified many avoidable risk factors leading to the improper use of inhaler devices among asthma patients visiting the ED.

## Introduction

Asthma is a chronic inflammatory disease of the airways associated with bronchial hyper-responsiveness and reversible airflow obstruction [1,2]. The incidence and prevalence of asthma have increased during the past 20 years, affecting 5-10% of the global population [3,4]. The prevalence of bronchial asthma among Saudi patients is approximately 20-25% [4,5]. The primary goal of asthma treatment is to control symptoms and to reduce emergency department (ED) use for acute asthma treatment [1,6-8]. One study reported only 5% asthma control among patients seen at tertiary care hospital [9]. Poor asthma control remains a frequent cause of ED presentation and hospital admission [10], and the cost of uncontrolled asthma care is substantial. For example, ED use for asthma management accounts for almost one-third of all asthma costs in the United States [11]. The administration of corticosteroids via inhalation is considered the optimal route for appropriate drug delivery for treatment of bronchial asthma and could reduce asthma hospitalizations by as much as 80% [12]. The most important advantage of inhaled therapy is the direct, localized delivery of a high concentration of drugs to the airways with minimal systematic side effects [13]. However, improper inhaler device use is one of the most common causes that hinder better asthma control [14-18].

The improper use of inhaled devices in the management of bronchial asthma decrease drug delivery, patient’s adherence to the treatment regimen and drug effectiveness. This subsequently leads to uncontrolled asthma management and multiple ED visits [14,15,17-22]. The improper inhaler device use as a cause of uncontrolled asthma management and frequent ED visits, to best of our knowledge, had never previously been studied in the Saudi population. The objective of this study was to evaluate the inhaler technique among asthmatic Saudi patients seen in ED and to identify the characteristics of these patients along with factors associated with the improper use of inhaler devices, asthma control and the number of ED visits.

## Methods

This cross-sectional study was conducted at the King Abdulaziz Medical City – King Fahad National Guard Hospital in Riyadh (KAMC-KFNGH) and the King Khalid University Hospital (KKUH). We enrolled adult patients (≥ 18 years old) diagnosed with asthma who visited the ED for asthma management between August 2010 and March 2011. The enrolled patients had a documented diagnosis of bronchial asthma as diagnosed by their primary physician and were on a prescribed inhaled corticosteroid (ICS) therapy for at least the last three months. We excluded patients without a documented diagnosis of bronchial asthma and those who were not prescribed ICS according to their medical records.

This study was approved by the Institutional Review Board (IRB) (Ref. IRBC/123/11). During the ED visit, a trained co-investigator collected information about demographic data, the duration of the illness and the medication used for asthma therapy. Additionally, the data were gathered on whether the patient received any formal education about asthma as a disease and, how to use their inhaler devices. The co-investigators also verified this information by reviewing the medical record of the patients and they assessed the asthma control over the last month by administering a validated published Arabic version of the Asthma Control Test (ACT) [23]. The co-investigators also determined whether the patient knew how to use the prescribed inhaler properly following specific steps in the check list (Table 1). All patients were observed for two trials of using their inhalers and proper use was identified if the patient fulfilled all of the steps required. The written informed consent was obtained from all participants.

**Table 1:** Inhaler device check list

	
Use of a pressurized metered-dose inhaler	
1.	Shake the canister
2.	Hold the canister upright at opening of mouth
3.	Begin a slow breath
4.	Actuate the MDI once while continuing with a slow breath
5.	Inhale to total lung capacity
6.	Hold breath for at least 4 seconds
MDI with spacer	
1.	Remove the cap of the spacer.
2.	Remove the cap of the puffer. Shake the puffer 5 or 6 times.
3.	Insert the puffer in the hole at the back of the spacer.
4.	Blow all your breath out until lungs are empty.
5.	Insert the spacer mouthpiece into the mouth
6.	Press the down once on the puffer's canister.
7.	Slowly breathe in from the spacer full breath.
8.	Hold your breath for at least 4 seconds
Using the turbuhaler	
1.	Unscrew the cover and remove it.
2.	Holding the Turbuhaler upright, turn the colored wheel one way and back the other way until it clicks.
3.	Breathe out normally.
4.	Put the mouthpiece between your lips and tilt your head back slightly.
5.	Breathe in deeply and forcefully.
6.	Hold your breath for 10 seconds
Diskus	
1.	Open the device
2.	Slide the lever
3.	Exhale away from device, to empty lung
4.	Place mouthpiece between teeth and lips
5.	Inhale rapidly and fully

### Statistical analysis

The data collected was transferred and analyzed using SAS® versions 9.2 (SAS Institute Inc., Cary, NC). Descriptive statistics, such as the means and standard deviations, were used to summarize the quantitative variables. The frequencies and percentages were used to summarize categorical variables. Chi-squared tests were used to test the association between clinical characteristics across the variables regarding asthma device use and asthma control test. P-values less than 0.05 were considered significant. Multiple logistic models were used to identify the risk factors that were associated with the improper use of asthma inhaler devices. The odds ratios (ORs) with 95% CIs were reported to describe the strength of these associations.

## Results

Among the 450 asthma patients, 176 (39.1%) were male (Table 2). The mean age was 42.3 ±16.7 and the mean duration of asthma was 155.9 ± 127.1 weeks. There were 270 (60%) patients with regular physician follow-up. Approximately half of the patients, 232 (51.6%) had no formal asthma education as a disease and 183 (40.7%) had no formal education about the medications or the asthma inhaler devices by any health care professional. Of the patients who received asthma and device education, 200 (44.5%) were educated by physicians, 35 (7.8%) were educated by asthma educators, and 21 (4.7%) were educated by the pharmacists. A total of 165 (36.7%) patients had three or more ED visits per year. Asthma control in the month preceding the ED visit (as per the ACT) was as follows: uncontrolled (ACT ≤ 15) was 105 (23.3%), partial control (16 ≥ ACT ≤ 23) was 335 (74.4%), complete control (ACT ≥ 24) was 8 (1.8%), and missing ACT 2(0.5%).

**Table 2:** Demographics and clinical characteristics about bronchial asthma (N=450)

**Characteristics**	**Levels**	**N (%) ^╦^**
Age, *(Mean ± SD)*		42.3 ± 16.7
Duration of illness in weeks, (*Mean ± SD*)		155.90 ± 127.13
Duration of illness, *n(%)*	*> 1 Year*	429 (95.97)
Gender, *n(%)*	*Female*	274 (60.9)
Marital status	*Married*	371 (83.6)
Improper use of asthma inhaler devices		203 (45.0)
Education level	*High school or less*	387 (86.0)
	*University*	62 (13.8)
	*Missing*	1(0.2)
Follow-up consistently with doctor		270 (60.0)
Follow-up clinic	*PHC/FM*	208(46.2)
	*Pulmonary*	46(10.2)
	*Internal Medicine*	8(1.8)
	*Others*	8(1.8)
	*No follow-up*	180(40)
No education about asthma		232(51.6)
No education about medication		183(40.7)
ER visits	*≥3*	165(36.7)
Asthma control	*Uncontrolled*	105(23.3)
	*Partially controlled*	335(74.4)
	*Complete control*	8(1.8)
	*Missing*	2(0.5)
Received health education about asthma disease from a physician		200 (44.5)
Received health education about asthma disease from a health educator		35(7.8)
Received health education about asthma disease from a pharmacist		21(4.7)
Knew about asthma disease independently		27(6.0)
Device	*MDI*	361(80.2)
	*Turbuhaler*	43 (9.6)
	*Diskus*	38 (8.4)
	*MDI with spacer*	3 (0.7)
	*Missing*	5 (1.1)

^╦^All percentages were rounded to one decimal place.

The improper use of asthma inhaler devices was observed in 203 (45%) of the patients. The improper use had a significant association with irregular clinic follow-ups, lack of education about asthma medication, lack of education about asthma as disease, uncontrolled asthma, and three or more ED visits (See Table 3). The patients with irregular clinic follow-up compared with regular follow-up were more likely to misuse the asthma device (60.9% versus 34.8%, p = 0.0001). Patients who received no education about asthma medication compared with those who did were more likely to use an asthma device improperly (54.6% versus 38.7%, p = 0.0009). Patients with uncontrolled ACT (score ≤ 15) compared to partially/fully controlled ACT (score > 15) were more likely to use asthma device improperly (59.1% versus 40.8%, p =0.001). Patients with 3 or more ED visits because of asthma exacerbations were more likely to improperly use an asthma device compared to those who visited less than 3 times (50.9% versus 41.3%, p =0.0497). Moreover, patients who were diagnosed with asthma for less than 1 year were more likely to use an asthma device improperly compared to those who were diagnosed for more than 1 year (77.8% versus 43.8%, p =0.005). Not receiving health education about asthma disease from a physician is associated with misuse of the device (57.4% versus 30.0%, p =0.0001). Also, our analyses show that this improper use of the device was not associated with gender, age, or education level (p > 0.05). After controlling for all other factors, four risk factors were found to be associated with improper use of the devices: uncontrolled asthma, irregular use of ICS, irregular follow up with clinic and lack of education about asthmatic disease (p < 0.05; Table 4). Additionally, we found patients who lacked asthma education were more likely to use the asthma device improperly compared with the group who received education (OR: 1.65; 95% CI: 1.07, 2.54). Patients who did not follow-up regularly with clinical appointments were also more likely to improperly use asthma devices than those who regularly followed-up (OR: 1.726; 95% CI: 1.081, 2.756). This study also revealed that patients with an uncontrolled ACT (score ≤ 15) were 7 times more likely to use inhaler devices improperly compared with patients with fully controlled ACT (OR: 7.414; 95% CI: 1.345, 40.857).

**Table 3:** The association of asthma device use with demographic and clinical characteristics

**Characteristics**	**Levels**	**Improper 203 (45%)**	**Proper 247 (55%)**	**P-value**
Gender	*Female*	123(44.9)	151(55.1)	0.9066
Age	*≥ 45 Years*	82(49.7)	83(50.3)	0.1369
Follow up with doctor	*Yes*	94(34.8)	176(65.2)	0.0001*
	*No*	109(60.9)	70(39.1)	
Educational level	*High school or less*	173(44.7)	214(55.3)	0.5884
Education about medication	*Yes*	103(38.7)	163(61.3)	0.0009*
	*No*	100(54.6)	83(45.4)	
Education about asthma	*Yes*	71(32.7)	146(67.3)	0.0001*
	*No*	132(56.9)	100(43.1)	
ACT	*Uncontrolled*	62(59.1)	43(41.0)	0.0010*
*Full/Partially controlled*		140(40.8)	203(59.2)	
ED visits	*≥ 3*	84(50.9)	81(49.1)	0.0497*
	*< 3*	114(41.3)	162(58.7)	
Duration of asthma	*>1 Year*	188(43.8)	241(56.2)	0.0046*
	*≤1 Year*	14(77.8)	4(22.2)	
Received health education about asthma disease from a physician				
	*Yes*	60(30.0)	140(70.0)	0.0001*
	*No*	143(57.4)	106(42.6)	
Received health education about asthma disease from a health educator				
	*Yes*	14(40.0)	21(60.0)	0.5188
	*No*	189(45.7)	225(54.4)	
Received health education about asthma disease from a pharmacist				
	*Yes*	6(28.6)	15(71.4)	0.1166
	*No*	197(46.0)	231(54.0)	
Knew about asthma disease independently				
	*Yes*	11(40.7)	16(59.3)	0.6302
	*No*	192(45.5)	230(54.5)	
Device	*MDI*	189(45.7)	225(54.4)	0.6958
	*Turbuhaler*	6(46.2)	7(53.9)	
	*MDI with spacer*	6(31.9)	13(68.4)	
	*Diskus*	2(50.0)	2(50.0)	

*The Chi-square/Fisher exact statistic is significant at the .05 level.

**Table 4:** The odds ratios with 95% CIs for risk factors associated with improper use of an asthma device

**Variable**	**Reference**	**Estimate**	**SE**	**P-value**	**OR**	**95% CI on OR**	
Intercept		−0.5351	0.2924	0.0673			
Uncontrolled ACT	*Full control*	0.8778	0.3177	0.0057*	7.414	1.345	40.857
Partially controlled ACT	*Full control*	0.2477	0.3005	0.4097	3.948	0.743	20.983
ICS regular	*Regular*	0.4322	0.1160	0.0002*	2.374	1.506	3.740
No education about asthma disease	*Education about asthma disease*	0.2514	0.1100	0.0223*	1.653	1.074	2.544
No follow-up with doctor	*Follow-up with doctor*	0.2730	0.1193	0.0221*	1.726	1.081	2.756

* Wald Chi-Square statistic is significant at the .05 level.

## Discussion

Previous studies have shown that the improper use of inhaler devices decreases drug delivery, patient’s regimen adherence and drug effectiveness contributes to uncontrolled asthma and multiple ED visits [14,15,17-22]. In this study, we tried to identify the relationship between improper inhaler device use, asthma control and number of ED visits. To the best of our knowledge, this is the first study in Saudi Arabia to examine the factors possibly leading to improper asthma inhaler use. We believe that this study has a sound methodology, being conducted by personal interview, and patient information was confirmed by reviewing medical records for each patient. A trained investigator confirmed the inhaler device use against a standard checklist. Similar to other studies, this study demonstrated that improper inhaler use is common in our population and results from avoidable causes. Furthermore, we demonstrated that improper inhaler device use is associated with poor asthma control and frequent ED visits [17-22]. Interestingly, improper asthma device use is mainly due to a lack of knowledge regarding asthmatic disease.

In this study, a majority (92%) of the patients were using metered-dose inhalers (MDI). This finding is consistent with Saudi Arabian practice for this disease, as most of the patients were seen at primary health care and family medicine clinics where the most common form of inhalers are MDIs. However, this should not be accepted as the cause for improper inhaler use. In fact, studies have shown that newer dry powder inhalers (DPIs) are not associated with an improved inhalation technique. Devices should be selected based on a patient’s acceptance and preferences [16]. Selecting a device based on the patients’ preference is cost effective in the long term, even if the device is more expensive than the standard devices [24]. However, studies have shown that good educational practice results in the proper use of MDI which will be more cost effective in the long-term [16,25,26].

Importantly, we found that 40% of the patients did not receive any formal education by any health care professionals regarding the proper use of inhaler devices. This was mostly due to a lack of asthma education programs. Almost half of our patients used asthma devices improperly, resulting in more visits to the ED due to subsequently poor asthma control. The major avoidable factors for improper device use were a lack of education regarding asthma as a disease and how the patient use inhaler device correctly. Therefore, our health care system should emphasize establishing asthma education programs to educate patients on asthma and its management, particularly regarding the use of inhaler devices. These asthma education programs require continuous effort to educate patients and their caregivers. Studies have shown that standardized asthma education programs, education focused on self-management and behavioral change improves inhaler device use, adherence to treatment and asthma control [27,28]. Studies have shown that almost 50% of the patients used the devices correctly and this improved to more than 80% after instruction regardless of the device being used [29,30]. In this study, approximately 59% of the patients received education about how to use the inhaler devices. The education was given by physicians in 44% of cases. However, 30% still improperly used the medication. Furthermore, asthma educators and pharmacists only educated approximately 6-7% of patients about the proper use of inhalers. Similar to other studies, there was no difference in the appropriate use of device stratified by patient age or gender [31].

One limitation of our study was the documentation of specific education that was given to the patients. We had to rely on the patients’ recollection of the education, as the education was not documented in the medical records. Additionally, we were not able to evaluate the quality of the teaching and how many educational sessions our patients received by health care professionals. We also had no background information on the psychosocial factors of this group of patients with poor inhaler device use, as this was beyond the scope of our study. Another limitation of this study was that we did not assess the side effects of improper inhaler use and how much this might contribute to poor compliance with medication, asthma control and ED visits. However, studies have shown that trained asthma educators, respiratory therapists and pharmacists are better qualified to teach patients than other health care providers [32,33]. We previously documented that only 5% of our patients seen at tertiary care clinics are completely in control of their asthma [9], and we also documented that many of our patients have a false belief and misconception about asthma pathophysiology and inhaled steroid use [34]. Also, in this study we only assessed the essential steps required for proper drug delivery. We did not score each step separately or count the number of errors or omissions. In addition to our previous studies [9,34], the finding of this study clearly demonstrates some limitations in our health care system. There is an urgent need for a national asthma education program at all level of medical care. We believe that the lack of an appropriate asthma education program in our system leads to improper device use, lack of the patient’s knowledge about asthma, false beliefs and misconceptions about ICS. These deficiencies result in poor asthma control and increased ED visits. This study was limited to two academic centers in the Riyadh-central region. Most likely it does not represent the asthma care at the national level; thus, there is a need for national epidemiological studies to assess different aspects of asthma management.

## Conclusion

This study shows that improper asthma inhaler technique is common among patients visiting ED in tertiary care centers in Saudi Arabia. This improper technique is associated with poor asthma control and frequent ED visits. The lack of appropriate asthma education is likely a major cause of improper device use. Furthermore, national asthma studies are necessary to explore this problem and to prospectively study the value of an interventional asthma education program to improve asthma inhaler device use and clinical treatment outcomes.

## Competing interests

The authors declare that they have no competing interests.

The authors declare that they have no financial competing interests.

## Authors’ contributions

**JH**: Reviewed the scientific literature pertinent to the research question. Wrote the proposal and responded to reviewer and IRB comments. Created data collection forms and drafted the first manuscript. **AA**: Performed all the statistical analysis and wrote the results section. **HA**: Supervised the data collection at KAMC. **SB**: Scientifically contributed to writing the proposal.**HR**: Supervised the data collection at KKUH. **KM**: Provided scientific expertise and operational guidance for data collection at KAMC and actively precipitated in contributing to writing the manuscript assigned by the PI. **MS**: Scientifically contributed to writing the proposal and study conduct at KKUH. All authors read and approved the final manuscript.
